# How to perform and interpret the lung ultrasound by the obstetricians in pregnant women during the SARS-CoV-2 pandemic

**DOI:** 10.4274/tjod.galenos.2020.93902

**Published:** 2020-10-02

**Authors:** Murat Yassa, Memiş Ali Mutlu, Erkan Kalafat, Pınar Birol, Cihangir Yirmibeş, Arzu Bilge Tekin, Kemal Sandal, Esra Ayanoğlu, Mahmut Yassa, Ceyhun Kılınç, Niyazi Tug

**Affiliations:** 1 University of Health Sciences Turkey, Şehit Prof. Dr. İlhan Varank Sancaktepe Training and Research Hospital, Clinic of Obstetrics and Gynecology, İstanbul, Turkey; 2 Ankara University Faculty of Medicine, Department of Obstetrics and Gynecology, Ankara, Turkey; 3 Selahaddin Eyyubi State Hospital, Clinic of Obstetrics and Gynecology, Diyarbakır, Turkey

**Keywords:** Lung ultrasound, pregnancy, SARS-CoV-2, COVID-19

## Abstract

**Objective:**

Evidence for the use of lung ultrasound scan (LUS) examinations in coronavirus 2019 pneumonia is rapidly growing. The safe and non-ionizing nature of LUS drew attention, particularly for pregnant women. This study aimed to contribute to the interpretation of LUS findings in pregnant women for the obstetricians.

**Materials and Methods:**

LUS was performed to pregnant women suspected of or diagnosed as having Severe Acute Respiratory syndrome coronavirus-2 (SARS-CoV-2) in the first 24 hours of admission. Fourteen areas (3 posterior, 2 lateral, and 2 anterior) were scanned per patient for at least 10 seconds along the indicated anatomical landmarks. The scan was performed in supine, right-sided and left-sided positions, respectively. Each area was given a score between 0 and 3 according to the specific pattern.

**Results:**

In this study, 21 still images and 21 videoclips that enabled dynamic and real-time evaluation were provided. Pleural line assessment, physiologic A-lines, pathologic B-lines, light beam pattern, white lung pattern, and specific patterns for quick recognition and evaluation are described.

**Conclusion:**

The potential advantages and limitations of LUS and its areas of use for obstetricians are discussed. LUS is a promising supplementary imaging tool during the SARS-CoV-2 pandemic. It is easy to perform and may be feasible in the hands of obstetricians after a brief didactic course. It may be a firstline imaging modality for pregnant women.

**PRECIS:** Lung ultrasound is feasible and relatively easy to perform for obstetricians to be used during the COVID-19 pandemic.

## Introduction

Chest computed tomography (CT) is the gold standard in the diagnosis of coronavirus diseases-2019 (COVID-19) pneumonia. The prominent features of COVID-19 are subpleural, ground-glass consolidative pulmonary opacities^(1,2)^. However, CT is relatively expensive, not feasible for monitoring and patients admitted to the intensive care unit, has an ionizing radiation nature and carries the risk of transmission during transportation^(3)^.

By contrast, lung ultrasound scanning (LUS) is easy to perform, has a non-ionizing nature, and has the advantages of bed-side application and thus is well suited for monitoring patients^(2,3,4,5)^. Its interpretation is accepted as relatively easy because it is mainly based on pattern recognition and provides real-time dynamic images^(5,6)^. LUS has been traditionally used by non-radiologists as an adjunctive imaging tool^(4)^. Pulmonologists, emergency medicine physicians, thoracic and cardiac surgeons often benefit from LUS in the management of traumatic conditions and intraoperative situations^(7)^. Obstetricians also use ultrasound liberally in their routine clinical practice. Practically, the examination of the maternal lungs immediately after obstetric sonographic evaluation could be feasible for obstetricians, basically to ascertain the presence or absence of normality and specific patterns, and thus to determine the need for further multidisciplinary management^(4)^.

The attenuation of sound waves by the lung and bone tissues limits the use of LUS in the diagnosis of central lung diseases; therefore, LUS mainly targets artifacts that originate from peri-pulmonary lesions to reach a diagnosis^(8)^. Changes in the lung parenchyma following COVID-19 pneumonia begin in the distal regions and progress proximally^(3)^. Lesions are mostly located in the posterior and inferior fields of both lungs^(8)^. This feature makes LUS non-inferior to CT in the pandemic setting compared with other respiratory disorders. The pathologic progression of pneumonia of COVID-19 provides credibility to a surface imaging modality such as LUS^(3)^. Herein, it was aimed to provide a didactical, pictorial review to assist obstetricians in the multidisciplinary management of pregnant women suspected or diagnosed as haing COVID-19 infection.

## Materials and Methods

In this educational, non-systematic pictorial review, all lung images and videoclips were obtained with a dedicated machine [Esaote S.p.a., Italy; Manufactured by: Eizo Nanao Corp., Model: EA720] for use in pregnant women with suspicion or diagnosis of COVID-19. A 1-8-MHz convex transducer was used on the regular obstetric preset.

**Figure 1:**
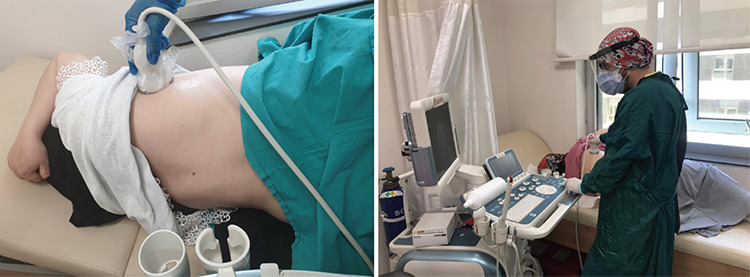
Lung ultrasound examination following a fetal assessment in supine and sided positions

Fourteen areas (3 posterior, 2 lateral, and 2 anterior) were scanned per patient for at least 10 seconds along the indicated anatomical landmarks^(9)^. The scan was performed in supine, right-sided and left-sided positions, respectively (Figure 1). Where applicable, scanning from the intercostal space was preferred.

Each area was given a score between 0 and 3 according to the specific pattern^(9)^. The pattern with a continuous and regular pleural line and horizontal artifacts, referred to as A-lines, was classified as score 0. The pattern with an indented pleural line and sporadic vertical white areas below the point of discontinuity in the pleural line, referred to as sporadic B-lines, was classified as score 1. The pattern with a broken pleura, small consolidated areas below the discontinuity, and multiple vertical white areas that reached the bottom of the field of view, referred to as multiple B-lines, was classified as score 2. The pattern with a severely broken pleura and a dense and largely extended white lung pattern with or without larger consolidations was classified as score 3. At the end of the procedure, the highest score obtained for each area was noted (e.g*.* landmark 1, score 0; landmark 2, score 1; and so on)^(5)^.

Local Instutional Ethical Board and National Scientific Research Board approved the study. Written consent was obtained from all patients underwent lung ultrasound.

## Results

In this study, six figures and three videos for score 0 (Figure 2, 3, 4, 5, 6, 7, Video 1, 2, 3), five figures and three videos for score 1 (Figures 8, 9, 10, 11, 12, Video 4, 5, 6), 5 figures and seven videos for score 2 (Figure 13, 14, 15, 16, 17, Video 7, 8, 9, 10, 11, 12, 13), four figures and four videos for score 3 (Figure 18, 19, 20, 21, Video 14, 15, 16, 17) were provided and explained in detail. In addition, four featured videos were added showing pleural effusion, the co-existence of scores 0 and 1, and perihepatic and pericardial effusions (Video 18, 19, 20, 21). The clinical characteristics and outcomes of the patients were not in the scope of this study and were therefore not presented.

### LUS Findings

The ribs and their posterior shadowing can be seen when the probe is positioned longitudinally. Transverse positioning of the probe on the intercostal spaces should be preferred, where applicable.

**Pleural line assessment:** Attention should be paid to the sliding, thickness, and irregularities (e.g. unsmooth, discontinuous or interrupted, indentation, broken pleura) of pleural line and subpleural effusion, if they exist.

The visceral and parietal parts of pleura slide over each other in backward and forward directions with respiratory movements called the normal sliding sign^(10)^. The sliding sign is absent in some clinical conditions such as pneumothorax.

The subpleural consolidations appear as an irregular hypoechoic area. Small patchy, strip or nodule consolidations can often be observed as a subpleural lesion.

In the COVID-19 infection, pleural thickening and subpleural effusion were found to be about 1-2 mm and 2-3 mm, respectively, which can change as the disease progresses^(8)^.

**A-lines:** These represent repetitive reverberation artifacts and commonly appear as horizontal, parallel lines at regular intervals^(7)^. These lines represent a normal inflated peripheral lung when combined with a normal pleural sliding sign^(4)^.

**B-lines:** These lines are well-defined vertical hyperechoic artifacts arising from the pleural line and reach the bottom of the screen^(4)^. These lines move with the pleural line during respiration and may erase A-lines^(7)^. Sporadic/coalescent or multiple B-lines can be seen and the density and combination of the pathologic signs may be correlated with the probability of disease^(11)^.

Sometimes, fewer than three B-lines between two adjacent ribs may be seen in 30% of normal lungs^(7,10)^. However, possible false-positive cases should also be approached with great caution in the pandemic settings and should be considered as a possible pathologic condition until proven otherwise.

There are also false vertical lines including C-, E- and Z-lines, which can commonly be mistaken for B-lines. However, for obstetricians working in the COVID-19 pandemic setting, discriminating those from pathologic B-lines may not be clinically relevant because they refer to specialist’s (such as radiologist and pulmonologist) considerations to differentiate from underlying diseases. In addition, they are mainly differentiated with B-lines concerning their synchronous movements with inspiration and expiration. Basically, an obstetrician should pay attention to the synchronized vertical lines that move with respiration.

**Light beam pattern:** A specific pattern that consists of a shining band-form artifact spreading down from a large portion of a regular pleural line and often has an on-off effect with respiration that may also have normal A-lines visible in the background^(11)^. This pattern was proposed to reflect the acute phase of ground-glass opacities during the early spread of the active COVID-19 pneumonia^(11)^.

**White lung pattern:** This pattern corresponds to the increased density of the lung parenchyma in which physiologic A-lines and other vertical artifacts including B-lines are erased^(4)^.

Pleural effusions and air bronchograms, which are the reflection of air-filled bronchus in the context of opacity are rarely seen in COVID-19 infections and should lead physicians to superinfections or other differential diagnoses^(12)^.

## Discussion

Obstetricians should be responsive during the Severe Acute Respiratory syndrome coronavirus-2 pandemic because they are the frontline physicians for the pregnant population^(13)^ and should be ready for the second wave or the next epidemics or pandemics caused by other viruses. The use of LUS for pregnant women in the hands of obstetricians can make a difference during such exceptional and critical situations^(14)^. This pictorial study can be used for the training of obstetricians in the pandemic setting and encourage the liberal use of LUS.

LUS cannot be a substitute for chest CT; however, it has certain advantages over CT as an adjunctive method in the diagnosis and management of respiratory involvement of COVID-19 infection, particularly for pregnant women^(4,5,8)^. The sensitivity and specificity of LUS in several clinical conditions range between 81% and 97%, and between 95% and 100%, respectively^(7,15)^. Authors postulate that LUS should be the first choice of imaging method in pregnant women suspected of having COVID-19 infection. However, LUS findings should be evaluated with the patient’s background because they are not always specifically attributable. More importantly, mild LUS findings (score 1) in an asymptomatic woman should be approached cautiously. For example, A-lines that are known as physiologic artifacts can represent abnormal signs in atelectasis, asthma, chronic obstructive pulmonary disease, and pneumothorax^(16)^. Similarly, B-lines can represent normal signs in healthy patients when they are fewer than three and do not reach the bottom of the screen^(16)^.

We have previously shared our clinical experience in eight cases showing that the use of LUS immediately after the fetal assessment can positively affect the clinical management of pregnant women infected with COVID-19^(5)^. As physicians without formal radiology residency training, we organized a brief course that consisted of a didactic lecture and hands-on ultrasound examinations supervised by experts^(17)^. This approach has been previously tested and found that LUS is feasible following theoretical training combined with still images taken from pregnant women infected with COVID-19^(6,18)^. The interobserver agreement between obstetricians with different levels of experience on still-images and videoclips of LUS was found as good^(17)^.

## Conclusion

LUS is a promising non-invasive, safe, and easily learned and performed imaging tool that can be used in pregnant women suspected of having COVID-19 pneumonia following an initial fetal assessment. This technical pictorial study can encourage the reasonable learning of LUS for obstetricians in the pandemic setting.

## Figures and Tables

**Figure 2:**
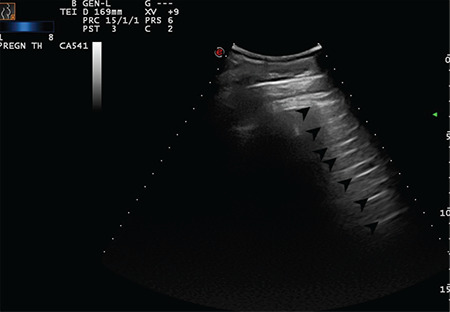
Regular pleural line. Arrows indicate physiological A-lines at regular intervals. Convex transducer positioned in the intercostal space (Scored 0)

**Figure 3:**
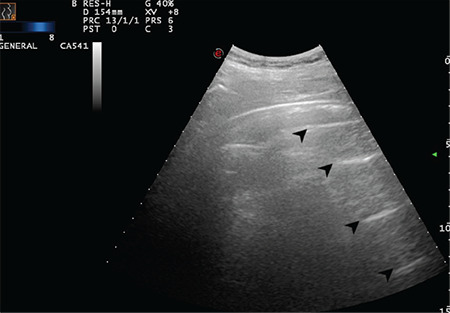
Regular pleural line. Arrows indicate physiologic A-lines at regular intervals (Scored 0)

**Figure 4:**
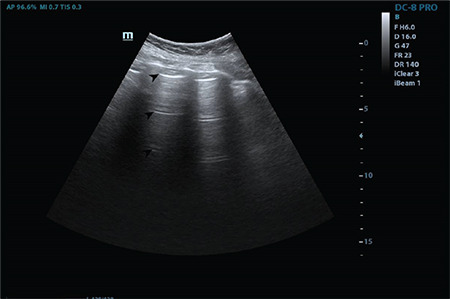
Normal LUS pattern with the convex transducer positioned longitudinally. Arrows indicate physiologic A-lines at regular intervals (Scored 0) LUS: Lung ultrasound scanning

**Figure 5:**
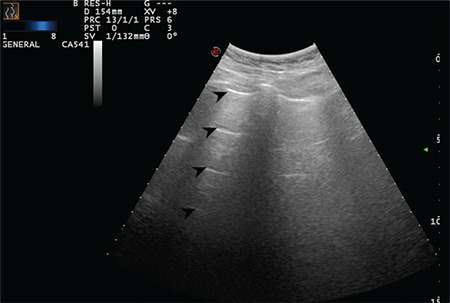
Normal LUS pattern with the convex transducer positioned longitudinally. Arrows indicate physiologic A-lines at regular intervals (Scored 0) LUS: Lung ultrasound scanning

**Figure 6:**
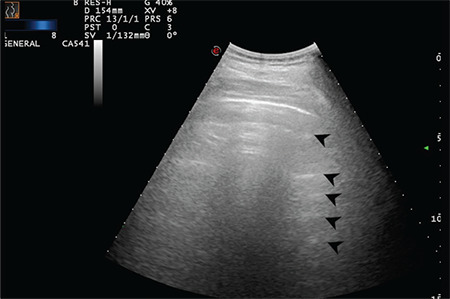
Regular pleural line. Arrows indicate physiologic A-lines at regular intervals (Scored 0)

**Figure 7:**
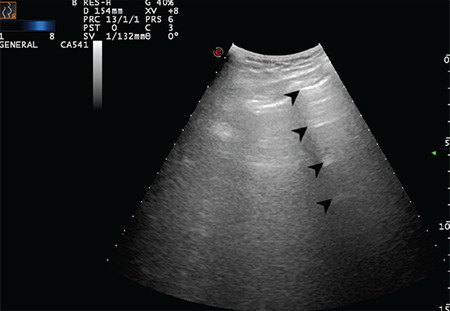
Regular pleural line. Arrows indicate physiologic A-lines at regular intervals (Scored 0)

**Figure 8:**
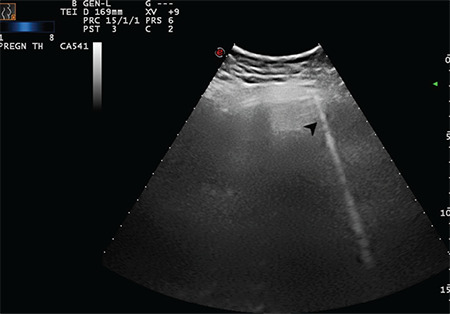
The arrow indicates a sporadic B-line arising from the intended pleural line (Scored 1)

**Figure 9:**
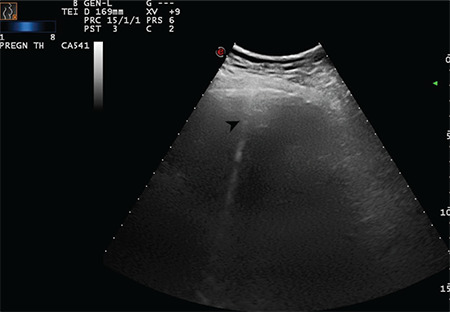
The arrow indicates a sporadic B-line arising from the intended pleural line (Scored 1)

**Figure 10:**
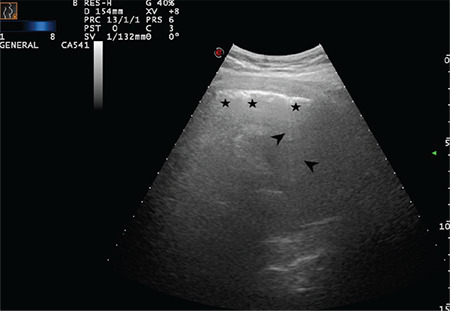
The arrow indicates a sporadic B-line arising from the intended pleural line. Stars indicate the thickened and intended pleural line (Scored 1)

**Figure 11:**
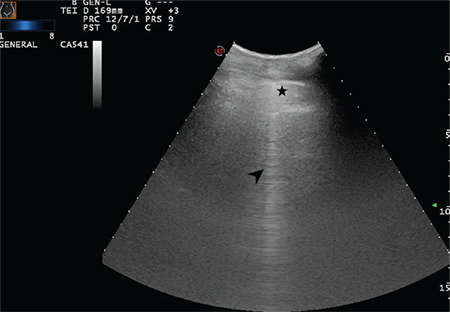
The arrow indicates a sporadic B-line arising from the intended pleural line. Star indicates the thickened and intended pleural line (Scored 1)

**Figure 12:**
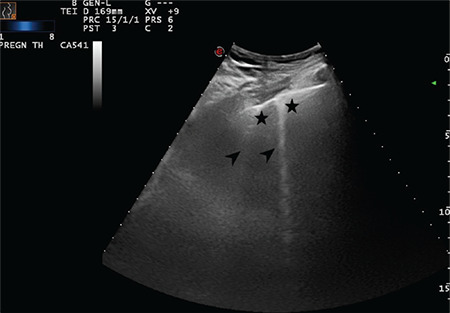
The arrow indicates a sporadic B-line arising from the intended pleural line. Stars indicate the thickened and intended pleural line (Scored 1)

**Figure 13:**
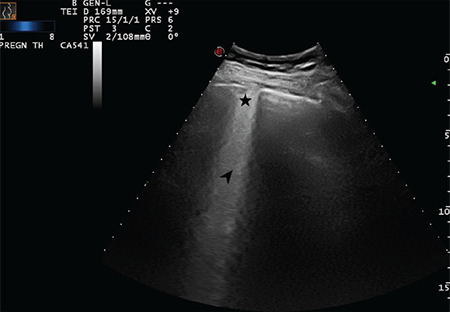
A broken pleural line and small consolidated area (indicated with star) below the irregularity and a large bright vertical area (indicated with arrow) can be seen (Scored 2)

**Figure 14:**
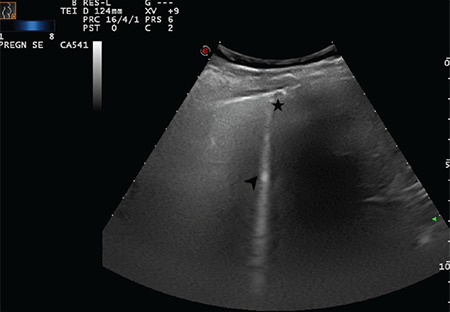
Star indicates a subpleural consolidation area and arrow indicate a B-line that reaches the bottom of the screen (Scored 2)

**Figure 15:**
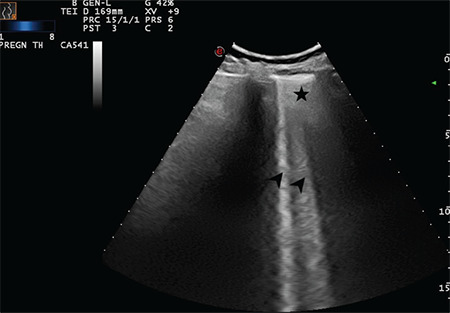
Arrows indicate multiple B-lines and the star indicates a broken and thickened pleural line (Scored 2)

**Figure 16:**
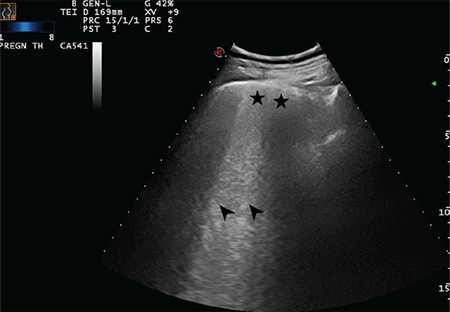
Arrows indicate multiple B-lines and stars indicate a broken and thickened pleural line (Scored 2)

**Figure 17:**
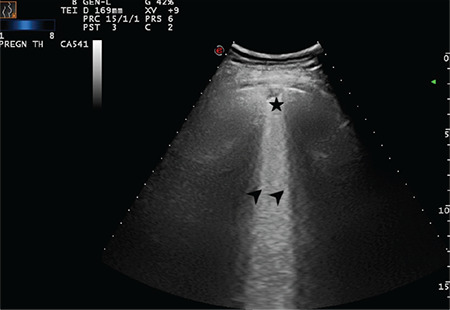
Arrows indicate multiple B-lines and star indicates a sub-pleural effusion and broken pleural line (Scored 2)

**Figure 18:**
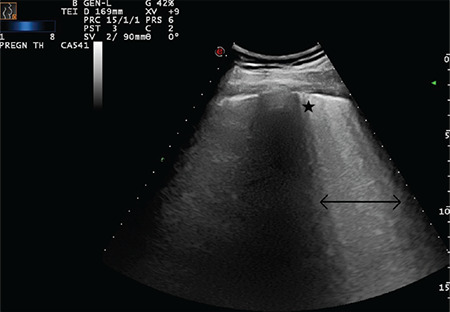
Double-headed arrow indicates a largely extended white lung pattern with small subpleural consolidation area as indicated with a star (Scored 3)

**Figure 19:**
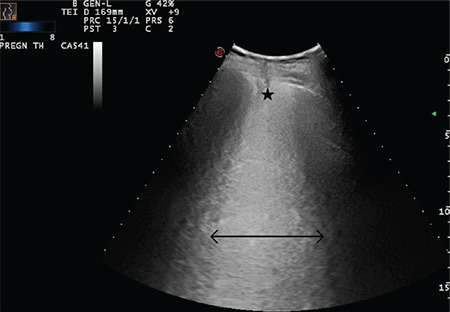
Double-headed arrow indicates a largely extended white lung pattern. The star indicates a severely broken pleural line and a small subpleural consolidation area (Scored 3)

**Figure 20:**
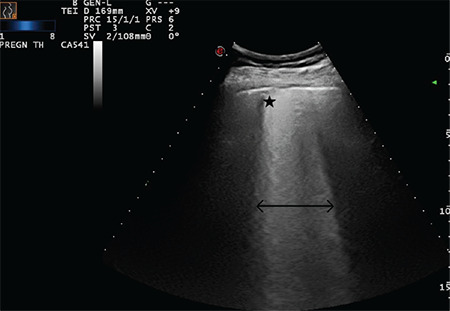
Double-headed arrow indicates a largely extended white lung pattern. The star indicates a severely broken pleural line and a small subpleural consolidation area (Scored 3)

**Figure 21:**
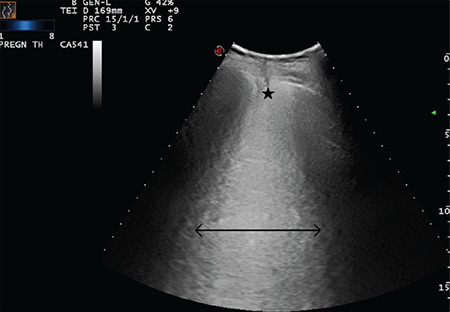
Double-headed arrow indicates a largely extended white lung pattern. The star indicates a severely broken pleural line and a small subpleural consolidation area (Scored 3)
